# Quality by design approach to optimize the formulation variables influencing the characteristics of biodegradable intramuscular *in-situ* gel loaded with alendronate sodium for osteoporosis

**DOI:** 10.1371/journal.pone.0197540

**Published:** 2018-06-01

**Authors:** Khaled Mohamed Hosny, Waleed Yousof Rizg

**Affiliations:** 1 Department of Pharmaceutics and Industrial Pharmacy, Faculty of Pharmacy, King Abdulaziz University, Jeddah, Saudi Arabia; 2 Department of Pharmaceutics and Industrial Pharmacy, Faculty of Pharmacy, Beni-Suef University, Beni-Suef, Egypt; 3 School of Pharmacy, boots science building, University of Nottingham, Nottingham, United Kingdom; Laurentian, CANADA

## Abstract

There are many challenges facing the use of alendronate sodium for the treatment of osteoporosis such as low bioavailability of 0.6% and oesophageal ulceration with bleeding. Due to the aforementioned limitation, the main objective of this research is to utilize a statistical experimental design in the formulation and optimization of alendronate in the form of controlled release biodegradable intramuscular *in-situ* gel. A Box–Behnken experimental design employing Statgraphics® software was used to develop an optimized *in-situ* gel formulation and to estimate the effects of Poly-DL-lactide-coglycolide as a primary polymer, the copolymer polycaprolactone, and lipid surfactant capryol 90. Every system was evaluated for gellation character, and *in vitro* release. As a novel technique for evaluation of the *in-situ* gel, *in-vivo* biodegradability rate was estimated in rats. Pharmacokinetic parameters were assessed in rabbits. The results indicated a significant effect of the copolymer and lipid surfactant on initial burst, and a significant effect of the primary and copolymer on drug percentage released. The optimum formulation showed a 5% initial burst, an *in-vivo* biodegradability rate estimated at 8% every seven days in rats, and the pharmacokinetic evaluation revealed that alendronate sodium mean residence time extended to 102 days in rabbits. In conclusion, the optimum biodegradable intramuscular *in-situ* gel formulations is a promising approach for providing higher bioavailability, extended release for more than three months, with elimination of esophageal side effects.

## 1. Introduction

Alendronate sodium (ALS) is an oral bisphosphonate that inhibits bone resorption due to its effect on osteoclasts. This inhibitory effect is potent and selective [1]. In all guidelines, ALS is recommended for the treatment of osteoporosis, either for postmenopausal women, or in patients with osteoporosis induced by a glucocorticoid [2]. The mechanisms of action of ALS include: an increase in bone density due to the bone turnover inhibitory action, and protection of the spine and hip by 50% through a decrease in the incidence of fractures [3].

However, there are many challenges to the use of this drug, including: low systemic bioavailability from commercially-available tablets, which is absorbed at about 0.6% after oral administration. This is due to the negative charges carried by its molecule, and at the same time, the very low lipophilicity of the molecules, which make its permeation across the gastrointestinal tract very difficult [4]. Another detriment to its use are esophageal side effects, such as esophagitis, ulcers, bleeding, and erosions [5]. As a result of the previously mentioned challenges, the drug is prescribed and used only under complicated precautions and instructions. Instructions include that the patient should remain standing for 0.5 h after ingestion of the drug, should ingest the tablet with a full glass of water, and not take any else for at least one hour after administration [6]. Several esophagitis cases have been reported due to patients not following the instructions properly [7]. As such, up to 60% of patients stop taking treatment ALS during the first year of therapy [8].

An *in-situ* gelling system is a type of drug delivery system that has several advantages. This type of drug delivery system is a solution under normal conditions *in vitro*, but undergoes a solution-gel transformation and converts to a gel structure. This transformation helps in attaining the required target during use [9]. Several applications of *in-situ* gel have been reported. These include controlled release drug delivery systems administered by oral, injectable, rectal, ocular, vaginal and intra-peritoneal routes [10]. Several mechanisms for solution-gel transformation have been reported for various types of polymer that were used in the formulation of the *in-situ* gel base. Transformation may occur due to a temperature change from room to body temperature, pH change from that of the preparation to the biological pH, and for some systems, the transformation occurs due to precipitation of polymers that are soluble in the solvent used in the formulation, but insoluble in body fluid [11]. *In-situ* gels are also utilized as a type of mucoadhesive drug delivery system. As such, there are several nasal applications that have utilized this approach to ensure intimate and prolonged contact with the mucous membrane lining the nasal cavity [12].

In contrast to strong gels, *in-situ* gel can be easily injected when administered through an intramuscular injection. After injection the gel swells and forms a strong gel in the muscle. This gel matrix is then capable of releasing a drug in a controlled manner with a prolonged residence time [9]. Biodegradable injectable *in-situ* gels represent an attractive implant that acts as a parenteral depot system as the drug release occurs over time as the polymer biodegrades [13].

The type, concentration, and molecular weight of the polymer usually affects the release rate of a particular drug [14]. The most common synthetic, degradable polymers are polyglycolide, polylactide, polyglycolide-*co*-lactide and polycaprolactone [15]. Most of these polymers degrade at a slow rate, which allows for the release of the loaded drug in a controlled manner over a prolonged period of time [16].

This research aimed to reformulate alendronate in the form of an *in-situ* gel as a controlled release depot intramuscular injection to be given every three months. This will enhance the bioavailability of alendronate and improve patient compliance as well as therapeutic benefits. A Box–Behnken experimental design was used to develop an optimized *in-situ* gel formulations that has low initial burst and adequate controlled release.

## 2. Materials and methods

### 2.1. Materials

Alendronate Sodium was kindly gifted by (Saja Pharmaceuticals Co. Ltd., Jeddah, Saudi Arabia). Potassium dihydrogen orthophosphate was purchased from BDH chemicals Ltd (Poole, England). Capryol 90 was kindly obtained from Gattefosse (France). N-Methyl-2- Pyrrolidone (NMP) was supplied from Acros organics (New Jersey, USA). Poly-DL-lactide-coglycolide and polycaprolactone were purchased from Sigma Chemical Company (St. Louis, Missouri, USA). All other chemicals utilized were of analytical reagent grade.

### 2.2. Methods

#### 2.2.1. Experimental design

Three different independent variables namely; concentration of PLGA (X_1_), PCL concentration (X_2_) and Capryol 90 concentration (X_3_) were studied to investigate their effect on the percentage of ALS release after 2 hrs (Y_1_) and after 24 hrs (Y_2_). A Box–Behnken experimental design was used to develop an optimized *in-situ* gel formulations that has a low initial burst and controlled release. The polynomial equations were obtained from the design in order to relate the independent variables with the responses (Y_1_and Y_2_). Statgraphics^®^ plus, version 4 (Manugistic Inc., Rockville, MD, USA) software was used in the experimental design of this study. The factor levels, codes (low, medium and high settings: −1, 0 and +1), and actual values of the different independent variables are given in Table 1. The levels of these values were optimized in order to obtain optimum dependent responses (Y_1_ and Y_2_).

**Table 1:** Coded and actual values for the independent variables selected to perform the optimization.

Level	Low	Medium	High
**Coded values**	-1	0	+1
**X**_**1**_^**a**^	10%	20%	30%
**X**_**2**_^**a**^	5%	10%	15%
**X**_**3**_^**a**^	1%	4%	8%

^a^ X_1_ is % of PLGA, X_2_ is % of PCL, and X_3_ is % of Capryol 90

Fifteen different formulations were prepared (According to the experimental design in Table 2) by dissolving 100 mg of alendronate sodium in 2 ml of N-methyl pyrrolidone containing a specific concentration of Capryol 90 according to coded values given in Tables 1 and 2. “For example the first formulation contain Capryol 90 with coded value equal -1 as appear in Table 2, this code value according to Table 1 equal 1% concentration”, and sonicated by using a probe sonicator. Specific amounts of PLGA polymer and PCL copolymer according to coded values given in Tables 1 and 2, were added and allowed to dissolve in a shaking water bath at 25°C for 7 days. The final solution was then stored in a refrigerator for later further evaluation.

#### 2.2.2. Evaluation of the *in-situ* gels formulations

pH and clarity and rheological properties: The pH of each formulation was measured by using a pH meter. The clarity was examined by visual inspection under a black and white background. The rheological behavior of each system was determined with a Brookfield viscometer before and after gelling. The shear rate varied between 0 and 300/sec and the corresponding shear stress was recorded. The gel strength is the share stress measured at low share rate after a mud has set quiescently for a period of time (10 second and 10 minutes in the standard procedures). The *gel strengths* were measured with the rotating viscometer. The sample stirred at high speed for 10 seconds, then allowed to stand undisturbed for 10 seconds. With the gears in neutral, a slow (about 3 rpm), steady motion on the hand wheel is applied. The maximum reading is the initial gel in pounds per 100 ft2. The mud is re-stirred for 10 seconds and allowed to stand for 10 minutes. The measurement is repeated as before and the maximum reading is recorded as the 10-minute gel strength in pounds per 100 ft2.

In-vitro release study: An *in-vitro drug* release study was performed in a phosphate buffer saline solution containing 0.02% sodium azide of pH 7.4 maintained at 37°C for 48 hr at 50 rpm using a USP dissolution tester apparatus II, with each formulation containing 100 mg of ALS. The samples were taken at different time intervals and analyzed for ALS content by a method proposed by Al Deep et al. 2004 [17]. The experiment was carried out in triplicate.

The *in-vitro* release data were analyzed by Statgraphics^®^ plus, version 4 (Manugistic Inc., Rockville, MD, USA) software to obtain a mathematical relationship between the independent and dependent variables. The optimized levels of X_1_, X_2_ and X_3_ that minimize both Y_1_ and Y_2_ were identified.

#### 2.2.3. Preparation and characterization of alendronate sodium *in-situ* gel optimized formulation

An optimized formulation containing an optimized level of each independent variable was prepared and *in-vitro* release performance was assessed as previously described. The observed values for Y_1_ and Y_2_ were calculated, compared with the values predicted by the design and the residual was estimated.

#### 2.2.4. The *in-vivo* biodegradability of the optimum *in-situ* gel formulation

Fifteen male Wistar rats, each weighing 200–250 g were used for the experiment. Rats were randomly divided into five groups of three rats each. The surgical procedures were revised and approved by the Animal Ethical Committee, Faculty of Pharmacy, King Abdulaziz University (Approval No. 321–2017 at 22.April.2017). The formulation was injected intramuscular in the left flank region of the rats at a dose of 1 mg/kg using a 20-gauge needle and the injection site was pressed between the fingers for a few seconds to prevent the liquid formulation from leaking out. At 1, 7, 14, 21, and 30 days, a group of rats were sacrificed by using an overdose euthasate, and the injection sites were incised, the implant detached, and its diameter and weight were measured.

#### 2.2.5 *In-vivo* pharmacokinetic study

Twelve healthy albino male rabbits weighing 2–2.5 kg were divided into two groups, with six animals each. The *in-vivo* animal studies protocol was revised and approved by the Animal Ethical Committee, Faculty of Pharmacy, King Abdulaziz University (Approval No. 334–2017 at 2.Jul.2017). Group 1 was injected intramuscularly with 1 mL of an optimized *in-situ* gel formulation in the range of 10 mg/kg body weight. Group 2 was injected with an alendronate sodium aqueous suspension. Blood samples of 0.5 ml were collected before administration and then at 1, 6, 12, 24 hours during the first day and after 3, 7, 14, 21, 30, 60, and 90 days from the first group, while blood samples were collected before administration and then after 1, 3, 6, 12, and 24 hours during the first day and after 3 and 7 days. The blood samples were centrifuge immediately after collection and stored at −20°C until the time of analysis.

#### 2.2.6. Pharmacokinetic analysis

Pharmacokinetic parameters were calculated and are presented as the mean ± S.D. The WinNonlin^TM^ Nonlinear Estimation Program was utilized to calculate the most important pharmacokinetic parameters as mean residence time and area under plasma concentration time curve.

#### 2.2.7. Statistical analysis

The results will be presented as mean ± SD calculated over at least three data points. The significance of the difference between the tested alendronate sodium optimized *in-situ* gel formulation and the reference was assessed by utilizing one-way analysis of variance (ANOVA) at a level of p ≤ 0.05 using the SPSS program.

## 3. Results

### 3.1. pH, clarity, rheological properties, and *in-vitro* release

Clarity of all the formulations was found to be satisfactory. The pH of the formulations was in the range of 6.7–7.3. The two main prerequisites of an *in-situ* gelling system are viscosity and gelling strength. Table 2 shows gel strength values, viscosity of all formulations before and after gellation.

**Table 2:** Various alendronate sodium *in-situ* gel formulations, responses, and gel strength values, and viscosities of all formulations before and after gellation.

Run	X_1_	X_2_	X_3_	Y_1_	Y_2_	Viscosity before gellation (cp)	Viscosity after gellation (cp)	Gel Strength
**1**	1	0	-1	17	21	2.87±0.31	18.27±2.87	38.02
**2**	-1	-1	0	19	40	1.74±0.11	12.53±2.61	25.55
**3**	0	1	-1	14	20	2.31±0.34	24.99±3.01	46.93
**4**	-1	1	0	15	25	1.45±0.15	23.66±2.01	45.62
**5**	1	-1	0	18	29	1.65±0.16	11.84±1.81	26.14
**6**	0	0	0	16	23	1.95±0.26	17.47±1.72	35.74
**7**	1	0	1	9	18	2.61±0.33	16.03±3.01	34.88
**8**	0	0	0	11	22	2.01±0.31	17.26±2.01	37.16
**9**	0	0	0	12	21	1.88±0.37	19.44±3.51	33.42
**10**	0	1	1	5	17	2.71±0.41	23.16±3.02	43.96
**11**	0	-1	1	19	27	1.65±0.36	10.37±1.51	27.14
**12**	1	1	0	12	16	3.87±0.21	25.43±1.21	46.43
**13**	-1	0	1	5	30	1.64±0.31	16.03±3.01	36.55
**14**	-1	0	-1	26	36	1.55±0.26	15.16±3.02	37.22
**15**	0	-1	-1	21	28	1.35±0.26	11.44±3.51	28.34

**Fig 1:**
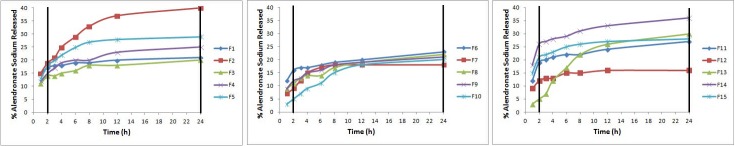
In-vitro release of alendronate sodium from different formulations.

Fig 1 shows the in-vitro alendronate sodium released from different prepared formulations at different times, the results of percentages released after 2 and 24 hours were taken as dependent variables in the optimization.

### 3.2. Polynomial equation and statistical analysis

The polynomial regression equations generated by Statgraphics^®^ plus, version 4 (Manugistic Inc., Rockville, MD, USA) software were as follows:

Y_1_ = 13.0–1.125*X_1_−3.875*X_2_−5.0*X_3_ + 1.25*X_1_^2^–0.5*X_1_X_2_ + 3.25*X_1_X_3_ + 1.75*X_2_^2^–1.75*X_2_X_3_ + 0.0*X_3_^2^

Y_2_ = 22.0–5.875* X_1_- 5.375* X_2_−1.25* X_3_+ 4.0* X_1_^2^ + 0.5* X_1_X_2_+ 0.75* X_1_X_3_+ 1.5* X_2_^2^ + 0.25* X_2_X_3_+ 0.25* X_3_^2^

Statistical analysis was performed using ANOVA, and the results are presented in Tables 3 and 4.

**Table 3:** Analysis of Variance for % Release after 2 hr.

*Source*	*Sum of Squares*	*Df*	*Mean Square*	*F-Ratio*	*P-Value*	Significance
A:PLGA %	10.125	1	10.125	0.73	0.4331	
B:PCL %	120.125	1	120.125	8.61	0.0325^a^	Significant
C:Capryol 90%	200.0	1	200.0	14.34	0.0128^a^	Significant
AA	5.76923	1	5.76923	0.41	0.5485	
AB	1.0	1	1.0	0.07	0.7996	
AC	42.25	1	42.25	3.03	0.1423	
BB	11.3077	1	11.3077	0.81	0.4092	
BC	12.25	1	12.25	0.88	0.3917	
CC	0.0	1	0.0	0.00	1.0000	

a: 0.001 ≤ p < 0.05

**Table 4:** Analysis of Variance for % Released after 24 hr.

*Source*	*Sum of Squares*	*Df*	*Mean Square*	*F-Ratio*	*P-Value*	Significance
A:PLGA %	276.125	1	276.125	37.06	0.0017^a^	Significant
B:PCL %	231.125	1	231.125	31.02	0.0026^a^	Significant
C:Capryol 90%	12.5	1	12.5	1.68	0.2518	
AA	19.07	1	19.07	7.93	0.1373	
AB	1.0	1	1.0	0.13	0.7291	
AC	2.25	1	2.25	0.30	0.6063	
BB	8.30769	1	8.30769	1.12	0.3393	
BC	0.25	1	0.25	0.03	0.8618	
CC	0.230769	1	0.230769	0.03	0.8672	

a: 0.001 ≤ p < 0.05

**Fig 2:**
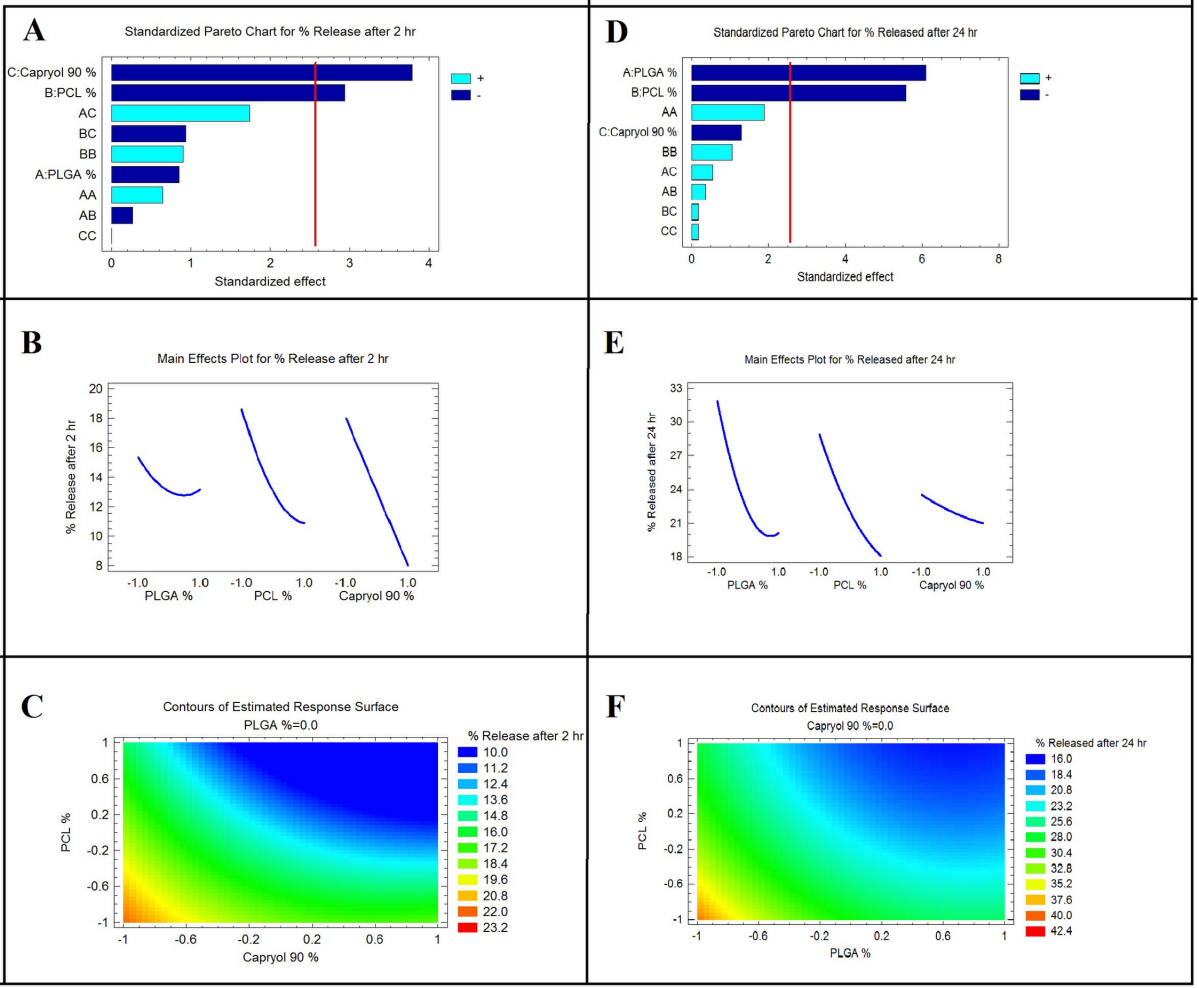
**A,** and **D** = Standardized Pareto charts for the effect of the studied variables on Y_1_ and Y_2_, respectively. **B** and **E** = Main effect plots for the effect of the studied variables on Y_1_ and Y_2_, respectively. **C** and **F** = Contour plots showing the relationship between various levels of the two significantly effects variables to attain fixed values of Y_1_ and Y_2_, respectively.

The relationship between the factors and responses can also be understood by plotting the response surface and contour for the estimated effects as shown in Fig 2.

### 3.3. Optimization of the formulation variables

An optimized alendronate sodium *in-situ* gel formulation with lower Y1 and Y2 values of 5.12% and 16.26%, respectively, was developed utilizing multiple response optimization by Statgraphics software. According to this optimization process, the predicted values of independent variables at maximum response desirability were 20% PLGA, 15% PCL, and 8% Capryol 90. The optimized *in-situ* gel formulation was prepared and subjected for *in-vivo* evaluation to assess biodegradability and pharmacokinetic behavior.

### 3.4. *In-vivo* biodegradability

**Fig 3:**
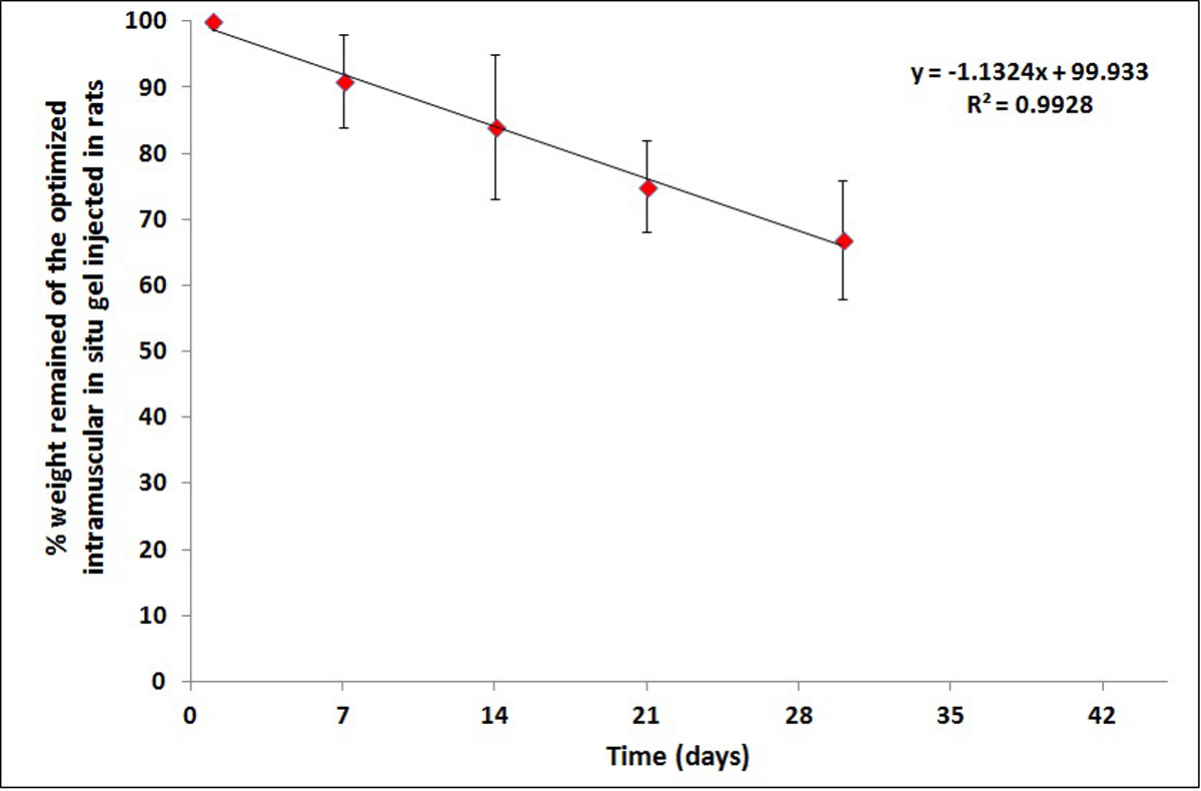
Relation between percent weight of implant remain intact inside the rat muscle and time intervals.

The results of *in-vivo* biodegradability indicated that the degradation of the optimized *in-situ* gel formulation was at a rate of 8% / 7 days. Biodegradability can also be understood by plotting the percent weight of implant remaining intact inside the muscle at different time intervals in rats (Fig 3).

### 3.5. *In-vivo* pharmacokinetic studies

The results of *in-vivo* pharmacokinetics indicated that the MRT for alendronate was extended to 102±8 days when administered as an *in-situ* implant in comparison to the MRT for an IM alendronate sodium aqueous solution which was only 4±2 days. The relative bioavailability of alendronate sodium was also optimized by the *in-situ* gel and was calculated according to AUC, which indicated an enhancement in bioavailability by more than 13 fold compared to the same dose of alendronate injected as an IM aqueous solution.

## 4. Discussion

The aim of this research was to reformulate alendronate in the form of an *in-situ* gel controlled-release depot intramuscular injection to be given every three months. This formulation would mitigate and address many of the challenges facing the use of alendronate sodium during the treatment of osteoporosis, such as low bioavailability 0.6–0.7% and oesophageal effects such as ulceration with bleeding.

A Box–Behnken experimental design was used to develop an optimized *in-situ* gel formulation with a low initial burst and subsequent controlled release. This experimental design was a response surface model used to hit the target, reduce variability in the experiment, and maximize/minimize a response that increases the production yield or decreases the amount of waste [18].

Polymers levels for the preparation of the *in-situ* gel were determined in order to result in a gel strength of between 25–50. Gel strength is the time-dependent forces in the drilling mud cause an increase in viscosity as the fluid remains quiescent for a certain period of time. It is a measurement of the electrochemical forces within the fluid under static conditions. Its field unit is the same as that of the yield strength [19]. Gel strength in the range between 25–50 was considered sufficient, and the gels that exhibited gel strength within this range could be readily administered. Gels with a strength greater than 50 s may be too stiff and cause discomfort [20]. The viscosities of the prepared gels were measured before and after gelation because the gel should have an optimum viscosity that facilitates injection as a liquid, and then undergoes a rapid sol-to-gel transition after injection, which facilitates sustained release of the drug and preserves its integrity without dissolving or eroding for a prolonged period of time [21]. One of the useful recent studies indicated the possibility of preparation of drug delivery systems based on smart silica gel in order to control the release of bisphosphonates [22].

Drug release after 2 hr was taken as an indicator of initial burst. The influence of individual factors indicated that capryol 90 concentration had a huge effect on percent released after 2 hr and that percentage dramatically decreased as the capryol 90 concentration increased. This could be because alendronate sodium is a highly water-soluble drug and capryol 90 was added to the formulations as lipid soluble surfactant in order to enhance the incorporation of the drug within the inorganic solvent [N-methylpyrollidone] used as a solvent for the biodegradable polymers [23]. Upon mixing of formulations with the aqueous dissolution media and the transition of formulations from solution to gel state, if the concentration of capryol 90 is low, alendronate will leave the formulations more easily and the initial burst increases. Therefore, an increase in the concentration of capryol 90 decreased the release of alendronate to the surrounding media. These results were confirmed by the significant negative effect of capryol 90 concentration on release after 2 hr obtained from the optimization data.

The negative significant effect of polycaprolactone concentration on the initial burst could be due to the increased viscosity of the system after sol-gel transition at a high PCL concentration, which decreases the ability of the alendronate sodium to leave the gel matrix, which decreases the inital burst. This was also confirmed by the viscosity measurements which indicated the high viscosity and gel strength of systems containing a high concentration of PCL compared to other formulations. These results agreed with the work of [24].

For alendronate sodium released after 24 hr, the influence of individual factors on this response indicated that by increasing the concentration of PLGA and PCL, the percentage released decreased. This may be because a higher polymer concentration reducing the amount of drug escaping into the external phase. These results agreed with the work of Ahmed et al. 2016 [25]. These results could also be due to the fact that PLGA and PCL degrade at a slow rate which ensures a controlled release pattern for the drug from the *in-situ* implant [26].

Biodegradability results indicated that the optimized *in-situ* gel formulation succeeded in prolonging the release of alendronate and confirmed the slow rate of biodegradability in the polymers used in the preparation. These results agreed with the work of [27]. Polymer composition is the most important factor to determine the hydrophilicity and rate of degradation of a delivery matrix which influence the rate of degradation. A systematic study of polymer composition with its degradation has been shown by many groups [28, 29]. These results show that increase in glycolic acid percentage in the oligomers accelerates the weight loss of polymer [30]. Thus absolute value of the degradation rate increases with the glycolic acid proportion.

The pharmacokinetic study confirmed that the bioavailability of ALS was enhanced by more than 13-fold when formulated as a controlled release *in-situ* gel compared to an intramuscular aqueous solution. The prolongation of action confirmed by the results of MRT were sustained for 102 days. This could be due to the slow degradation rate of biodegradable polymers used in the formulation [31]. This may also be due to the controlled release of alendronate from the formulations. In addition, the concentration of alendronate sodium in the peripheral compartments of the body leads to prolongation of terminal half-life, which sustains the MRT of the drug [32, 33].

## 5. Conclusion

A formulation utilizing alendronate sodium as an optimized controlled-release intramuscular *in-situ* gel as a novel drug delivery system provided a minimum initial burst and controlled release that eliminated undesired adverse drug effects. The *in-vivo* biodegradability rate was 8% every seven days which ensured a slow rate of degradation for the polymers, and enhanced the bioavailability of alendronate by more than 13-fold in relation to an intramuscular aqueous solution. The results indicated successful use of the Box-Behnken design developed thru Statgraphics® software and the prediction of a formulation for optimizing alendronate sodium in an *in-situ* gel. The developed novel formulation is promising for eliminating oesophageal inflammation and/or bleeding associated with the use of conventional tablets.

## Supporting information

S1 File Release data. (XLS)

S2 File Certificate for language editing. (PDF)
