# Supplementary material for: Quality by design approach to optimize the formulation variables influencing the characteristics of biodegradable intramuscular in-situ gel loaded with alendronate sodium for osteoporosis
Source: PLoS One. 2018 Jun 1;13(6):e0197540. doi: 10.1371/journal.pone.0197540 (PMC5983444; doi:10.1371/journal.pone.0197540)
Supplement: S2 File — (PDF) [file pone.0197540.s002.pdf]

SERVICESCAPE

Editing Certification

33 Pasho Street
Andover, MA 01810
United States
(978) 475-1713

Invoice Number: 353052
Certification Date: 3/12/2018

To whom it may concern:

This letter shall serve as an official certification of professional editing and proofreading services. The document detailed below was edited by an experienced and well-qualified English editor.

Title:	Alendronate In Situ Gel
Author:	khaled Hosny
Editor:	David Wong
Language:	English

This document is certified to have been edited for proper language, style, punctuation, spelling, and grammar. Information regarding the editor's qualifications can be found at https://www.servicescape.com/editors/doctorword.

The purpose of this certification is to declare the correctness of the edited document only. ServiceScape does not guarantee that the document is genuine or that the statements contained in the document are true. Furthermore, ServiceScape Incorporated assumes no liability for the way in which the edited document is used by the client or any third party, including end users of the edited document. ServiceScape Incorporated's limitation of liability applies to this certification.

Please contact us if you have any questions.

Best Regards,

David Costello

David Costello
CEO and Administrator
ServiceScape Incorporated
info@servicescape.com
https://www.servicescape.com
